# The Use of Horse and Donkey Meat to Enhance the Quality of the Traditional Meat Product (Kaddid): Analysis of Physico-Chemical Traits

**DOI:** 10.3390/foods13182974

**Published:** 2024-09-19

**Authors:** Mohamed Aroua, Nour Elhouda Fehri, Samia Ben Said, Alda Quattrone, Stella Agradi, Gabriele Brecchia, Claudia Maria Balzaretti, Mokhtar Mahouachi, Marta Castrica

**Affiliations:** 1 Université de Jendouba, Ecole Supérieure d’Agriculture du Kef, LR: Appui à la Durabilité des Systèmes de Production Agricoles du Nord-Ouest, Complexe Universitaire Boulifa, Le Kef 7119, Tunisia; arouamohamed2310@gmail.com (M.A.); sabensaid@gmail.com (S.B.S.); taymallahmah@gmail.com (M.M.); 2 Department of Veterinary Medicine and Animal Sciences, University of Milan, Via dell’Università 6, 26900 Lodi, Italy; alda.quattrone@unimi.it (A.Q.); gabriele.brecchia@unimi.it (G.B.); claudia.balzaretti@unimi.it (C.M.B.); 3 Department of Veterinary Sciences, University of Turin, Largo Paolo Braccini 2, 10095 Grugliasco, Italy; stella.agradi@unito.it; 4 Department of Comparative Biomedicine and Food Science, University of Padova, Viale dell’Università 16, 35020 Legnaro, Italy

**Keywords:** dried meat, physico-chemical traits, fatty acid profile, donkey and horse meat quality

## Abstract

The aim of this study was to evaluate the use of horse and donkey meat in the production of kaddid—a traditional dish typically not made with these meats—from a physical and chemical perspective. The results showed that both meats exhibit similar water retention during cooking, contributing to comparable tenderness and juiciness, with no significant differences in pH values, indicating similar quality (*p* > 0.05). However, their amino acid profiles differ: horse meat contains lower levels of glutamate (*p* < 0.05), methionine (*p* < 0.01), isoleucine (*p* < 0.05), and leucine (*p* < 0.05), but higher levels of proline (*p* < 0.05), histidine (*p* < 0.01), and lysine (*p* < 0.001) compared to donkey meat. Both meats provide essential amino acids. Horse meat is richer in saturated and monounsaturated fatty acids (32.44% and 39.58%, respectively), while donkey meat has a higher content of polyunsaturated fatty acids (31.51%), with a more favorable PUFA/SFA ratio, suggesting better cardiovascular health benefits. In terms of dried meat, donkey kaddid has a higher protein (17.45 g/100 g) and lower fat content (2.1 g/100 g) compared to horse kaddid (16.7 g/100 g, and 3.5 g/100 g, respectively) (*p* < 0.05). These findings inform consumer choices and production practices, promoting the use of horse and donkey meat for kaddid production.

## 1. Introduction

Despite shifts in lifestyles, changes in economic systems, and growing curiosity for culinary dishes that are diverse and distant from traditional practices [1], traditional culinary preparations remain central to defining cultural identities. The industrial-scale production and marketing of traditional culinary products would not only preserve and promote cultural heritage but also contribute to the growth of the agricultural and agri-food sectors in developing countries [2].

The dried and salted meat product known as kaddid or El Guedid is a traditional meat by-product commonly made from mutton and dried outdoors, primarily in the Maghreb countries of Tunisia, Algeria, and Morocco [3]. Kaddid can be stored at room temperature for over a year, and before consumption, it is desalted by soaking in water and then used in a variety of dishes [4]. In addition to kaddid, there are various other dried and fermented or non-fermented products made from sheep and goat meat in North Africa and the Mediterranean region [3]. The practice of making kaddid dates back centuries and has its roots in ancient times when communities needed to preserve meat without modern refrigeration. This method was particularly useful in arid climates, where the hot sun and dry air could naturally preserve food [5].

To date, advances in processing techniques, an increasing focus on food safety, and the incorporation of diverse or alternative ingredients have enhanced the safety, nutritional, and sensory qualities of these traditional products [6]. Several studies have examined the quality of kaddid, typically prepared by Northerners using sheep and cow meat and by Southerners using camel meat [2,7]. However, the quality of kaddid made with horse or donkey meat has not been broadly explored to date. Meat and meat products, in general, represent an important source of nutrients that may offer significant health benefits [8].

Donkeys (*Equus asinus*) are among the most significant domestic animals in many North African and Middle Eastern countries [9], valued for their ability to survive and reproduce under challenging environmental conditions. Today, donkeys serve multiple purposes. They are not only used as pack animals but also engaged in agriculture and utilized for dairy and meat production [10]. Donkey meat, though less familiar to many consumers, is known for being particularly lean, with a lower fat content than horse meat [11]. This characteristic can make it an attractive option for those seeking to reduce their intake of saturated fats and cholesterol. Additionally, the unique lipid profile of donkey meat, including a higher proportion of polyunsaturated fatty acids (PUFAs), has been linked to potential cardiovascular benefits [12,13,14,15]. Donkey meat is considered quite tough and is primarily used in processed products such as salami or other salted meat products, as discussed by Charqui [16] and Tasajo [17].

Regarding horse meat (*Equus caballus*), traditionally, older animals previously used for farm work were used for meat production [18]. However, today, horses are specifically bred for meat production [18,19]. Horse meat is appreciated in many cultures for its rich flavor and high iron content, which can help address iron deficiency anemia [20,21], a common nutritional concern. It is frequently incorporated into traditional dishes in parts of Europe, Asia, and Latin America. Studies indicate that horse meat is relatively high in protein and has a favorable fatty acid composition compared to more commonly consumed meats [11,21,22].

The aim of this study was to assess the physico-chemical quality and characteristics of donkey and horse meat to evaluate their suitability for kaddid production, with the goal of enhancing the nutritional, organoleptic, and sensory qualities of this traditional dish. This knowledge can guide consumers’ food choices and inform production practices to promote the use of horse and donkey meat in preparing this traditional dish. Additionally, it contributes to improving and enhancing its physico-chemical and organoleptic qualities.

## 2. Materials and Methods

### 2.1. Animals and Meat Sampling

Sixteen male donkeys, averaging 4 years old (±0.92), and sixteen Arab-Barb breed male horses, averaging 5 years old (±0.54), were included in the study. The donkeys, belonging to the Masri population, were raised together for 2 years under a previously described breeding system [15], this system provided them with unrestricted access to water and a daily ration of 1 kg of barley per animal. The donkeys were also grazed on grass pastures located in a mountainous region. The horses were raised together intensively for 3 years at a ranch in Bizerte, where they were fed concentrates and hay post-weaning during winter and grazed on pasture along with concentrates during summer. The concentrates consisted primarily of cereal grains, including oats, barley, and corn.

For this study, both donkeys and horses were transported to Ellouhoum Company, a slaughterhouse supervised by the Tunisian Ministry of Trade and Export Development. The animals were fasted for 12 h prior to slaughter, which was carried out by trained personnel in accordance with animal welfare standards set by national regulations (Law No. 2005-95, dated 18 October 2005). The slaughter procedures were designed to ensure animal welfare and adhered to the ethical guidelines established by the Institution de la Recherche et de l’Enseignement Supérieur Agricole of Tunisia.

The live weights of the horses and donkeys were recorded at the slaughterhouse. The carcasses were then weighed and stored in a cold room at 4 °C.

After 24 h, the cold carcass weights were measured, and cold dressing percentages were determined. Muscle samples, approximately 700 g in weight, were taken from the *Longissimus thoracis* (LT) on the right side of each donkey’s carcass, located between the 9th and 13th ribs. For horses, the samples were collected at the level of the 13th–14th thoracic vertebrae. To maintain freshness, around 350 g of each sample were vacuum-sealed and stored at 4 °C, and then immediately transferred to the laboratory for analysis at 48 h postmortem, while the remaining 350 g was used to prepare the kaddid meat.

### 2.2. Kaddid Preparation

For each animal, 350 g of meat from the LT was obtained and carefully sliced. Donkey and horse meat samples were seasoned and thoroughly mixed with a blend of powdered spices.

The spices used were garlic powder, coriander powder, salt, and paprika in respective quantities of 3 g, 4 g, 1.5 g, and 0.5 g per 100 g of meat. The meat was kept at room temperature (25 °C) for 12 h. After seasoning, the slices were dried using the natural sun-drying method. Meat strips were hung on a wire during the day and covered with mosquito nets to prevent insect infestation. Samples were collected daily before sunset and stored overnight in a cool, ventilated place until they reached a water activity (aw) of 0.57, indicating the end of drying. The kaddid was then stored in plastic boxes at room temperature for 50 days.

### 2.3. Physico-Chemical Analyses

The pH measurements at 24 h post-mortem were performed directly in the slaughterhouse by performing a penetrating electrode connected to a portable pH meter (model HI 98107 pHep, HANNA Instruments, Carrollton, TX, USA), calibrated with pH 7.01 and pH 4.01 buffer.

Upon the samples’ arrival at the laboratory, 48 h after slaughter, a 100 g of meat was taken from each LT sample for analysis of its chemical composition. The total protein content (crude protein, N × 6.25) was determined using the Kjeldahl method, as outlined in AOAC 928.08 [23]. The ash content was measured by mineralization at 550 °C according to AOAC 920.153 [23]. The intramuscular fat content was determined using the AOAC Official Method 991.36, titled “Fat (Crude) in Meat and Meat Products” [23]. The extraction of total lipids was conducted with a hot treatment using petroleum ether as the solvent. Moisture, protein, fat, and ash contents were assessed using methods specified by the International Organization for Standardization (ISO) [24]. Simultaneously, water content in 5 g minced meat samples was determined by drying in an oven at 105 °C, following the AOAC 950.46 standard [23].

In addition, after 48 h, colorimetric coordinates on donkey and horse meat LT samples were also evaluated. A Chroma Meter (model CR-400, Konica Minolta Holdings, Japan) was used, featuring a measured area of 8 mm, a 10° view angle, and a D65 illuminant.

Color measurements were taken on the exposed meat surface after 30 min of blooming at room temperature (20 °C) using the CIE system. The results represent the average of three readings. Chroma (C*) and hue (h*) values were derived from the measured lightness (L*), redness (a*), and yellowness (b*) values using the following formulas: h* = arctangent(b*/a*) and C* = √(a^2^ + b^2^). To obtain three distinct reflectance readings, the measuring lens was rotated through 0°, 45°, and 90° (clockwise) across the meat surface, and the measurements were averaged.

Cooking loss (*CL*) was determined according to the method described by Yagoubi et al. [2]. Uniform meat samples were initially weighed (*Wi*: initial weight) and placed in plastic bags suitable for cooking and which are heat-resistant. The samples were placed in a water bath at 75 °C and heated for 30 min until they reached an internal temperature of 75 °C, which was monitored with a thermocouple. After cooking, the bags were cooled under running tap water, and the meat was patted dry with paper towels. The cooked samples were then weighed again (Wf: final weight). *CL* was determined as the percentage difference between the samples’ initial and final weights, using the following formula:

(1) %CL=100×(Wi−Wf)/Wi

The Water-Holding Capacity (*WHC*) was determined following the method described by Bowker et al. [25], a 10 g sample of minced meat was combined with 15 mL of 0.6 M NaCl and mixed for 2 min. The mixture was then refrigerated at 4 °C for 15 min. After refrigeration, the mixture was shaken and centrifuged at 7669× *g* for 15 min. The *WHC* was calculated using the following formula:

%*WHC* = [(0.6 M NaCl volume − supernatant volume)/sample weight] × 100 (2)

Regarding the kaddid meat samples, only fat, protein, ash, color, and pH were analyzed.

### 2.4. Determination of Total Amino Acid Profile

For the determination of the total amino acid profile, the donkey and horse meat sample and kaddid were freeze-dried and subsequently hydrolyzed using 6 M hydrochloric acid (HCl) for 22 h at 110 °C. Prior to this step, cysteine and methionine were oxidized to cysteic acid and methionine sulfone, respectively. After hydrolysis, the resulting liquid (hydrolysate) was filtered through a 0.45-μm filter to eliminate any residual solid particles.

The filtered supernatants were then utilized for amino acid analysis. A High-Performance Liquid Chromatography (HPLC) system (Biochrom 30 series AA analyzer, Biochrom Ltd., Cambridge Science Park, UK) equipped with a resin exchange column (20 × 0.46 cm inner diameter) was employed to determine the amino acid profiles. Peaks were identified by matching their retention times with those of standard amino acids.

### 2.5. Analysis of Fatty Acid Composition

The fat content of donkey and horse meat samples and kaddid (after 50 days of aging) was extracted using the chloroform/methanol extraction method by Folch [26]. A fatty acid analysis was conducted by direct trans-esterification to methyl esters, following ISO 5509:200 standards [27]. Samples’ fatty acid compositions were analyzed using a capillary gas chromatograph (AGILENT 6890 N, USA) equipped with a flame-ionization detector and an HP-88 fused-silica capillary column (100 m × 0.25 mm × 0.2 µm film thickness). The chromatograph operated with a temperature program starting at 100 °C for 3 min, ramping up to 240 °C at 5 °C per minute, and holding at 240 °C for 10 min. Hydrogen was used as the carrier gas at a flow rate of 1 mL/min. The detector and injector temperatures were set at 260 °C and 255 °C, respectively, with a 50% split ratio. Fatty acids were identified by comparing their retention times with standard fatty acids.

The atherogenic index (*AI*) and thrombogenic index (*TI*) were computed according to the methodology outlined by [28] as follows:

*AI* = (C12:0 + (4 × C14:0 + C16:0))/(MUFAs + PUFAs) (3)

*TI* = (C14:0 + C16:0 + C18:0)/[0.5 × MUFAs) + (0.5 × n-6) + (3 × n-3) + (n-3/n-6)] (4)

where C12:0 is lauric acid, C14:0 is myristic acid, C16:0 is palmitic acid, C18:0 is stearic acid, n-6 refers to omega-6 fatty acids, n-3 refers to omega-3 fatty acids, MUFAs are monounsaturated fatty acids, and PUFAs are polyunsaturated fatty acids.

### 2.6. Statistical Analysis

The data were subjected to Student’s *t*-test to assess differences in meat chemical composition, cooking loss, color, water holding capacity, amino acid, and fatty acid composition between horse and donkey meat, as well as their respective kaddid preparations, additionally to the comparison between raw meat and spiced products by species (horse and donkey). The effect of the drying process on the kaddid produced from horse and donkey meat was analyzed using a one-way analysis of variance (ANOVA). The results were presented as least squares mean values with their respective standard errors. Statistical significance was defined as *p* < 0.05 using XLSTAT software (Addinosoft 2016) [29].

## 3. Results and Discussion

### 3.1. Carcass Characteristics

The data in Table 1 present a comparative analysis of body weight, cold carcass weight, and cold dressing percentage between the North African donkey population and the Arab barb horse breed.

Compared to donkeys, the Arab-Barb horse breed demonstrates significantly higher final body weight, cold carcass weight, and cold dressing percentage (Table 1). Remarkably, the cold dressing percentages of Tunisian donkeys and horses align with the reported values for other Mediterranean and European breeds, such as the Martina Franca breed and the Masri population for donkeys, as well as Polish horses [30].

Polidori et al. [12] noted that the cold dressing percentage is influenced by several factors, including the stage of maturity, degree of finishing, breed, and intestinal content.

### 3.2. Physico-Chemical Characterization of Asinine and Horse Raw Meat

The qualitative characteristics of North African donkey and Arab-Barb horse meat are presented in Table 2.

The technological parameters for both types of meat are similar. Both species exhibit a high capacity to retain water during cooking, contributing to their tenderness. Lower water loss is often associated with juicier and more tender meat, as it indicates higher liquid retention during cooking [15,31,32,33].

The pH values of both donkey and horse meats show similarities and reveal no significant differences (*p* > 0.05). The pH is crucial in meat preservation and processing [34]. This variation in pH is attributed to low muscle glycogen levels at slaughter, resulting in low lactate production and, consequently, tender meat. Higher pH values in horse meat, as compared to lamb meat, may also indicate proteolysis processes influenced by enzymes from microorganisms responsible for maturation [15,35,36]. The type of finishing diet and farming system can influence equine meat pH [13,37,38,39]. Factors such as pre-slaughter stress and fasting duration significantly impact meat quality. Increased pre-slaughter stress, animal fatigue during transport, and depletion of muscle glycogen stores result in higher meat pH [40,41,42].

Color is essential in meat quality perception and consumer purchasing decisions. It is an indicator of product freshness [43,44,45]. The lightness index (L*) is significantly higher for donkey meat than horse meat (*p* < 0.05). Redness (a*) and yellowness (b*) values differ significantly between donkey and horse meat (*p* < 0.05). These differences are attributed to variations in myoglobin concentration, species characteristics, and dietary factors [21,33,35]. Equine meat is distinguished by its darker brown color and lower lightness value compared to lamb meat, which exhibits a lighter color [46]. This characteristic can be attributed to the oxygenation of myoglobin, linked to the a* value, as equine meat has higher myoglobin concentrations in adulthood [21,33,35].

Donkey meat has slightly higher protein content compared to horse meat (*p* < 0.01), suggesting that donkey meat could be a better source of essential proteins. Additionally, donkey meat has a lower fat content than horse meat (*p* < 0.05), making it more attractive to consumers mindful of their fat intake. In contrast, ovine meat has a higher fat content (>2.5 g/100 g) than equine meat, though their protein levels are comparable range: 19 to 22 g/100 g) [47,48].

These variations may be explained by morphological differences between the species and breed, affecting fat and muscle tissue distribution and leading to differences in fat and protein content, as well as to breeding and feeding practices [49,50]. The North African donkey population, prevalent in Tunisia and raised in mountainous and border areas, may have access to diverse diets, including a variety of plants, herbs, and shrubs, increasing fat and protein levels in donkey meat. Conversely, horses might be subjected to different breeding and feeding practices, leading to varying fat, dry matter, and protein content [13,15,51,52].

### 3.3. Amino Acid Profile of Donkey and Horse Raw Meat

The total amino acid composition in North African donkey and Arab-Barb horse meat after HCl hydrolysis is reported in Table 3. Compared to donkeys, horse meat has significantly lower glutamate (*p* < 0.05), methionine (*p* < 0.01), isoleucine (*p* < 0.05), and leucine (*p* < 0.05) levels, but higher proline (*p* < 0.05), histidine (*p* < 0.01), and lysine (*p* < 0.001). Despite these differences, both types of meat provide all essential amino acids necessary for adequate nutrition, particularly beneficial for specific population groups like children, the elderly, and individuals with health issues.

Lysine emerges as the most abundant essential amino acid in equine meat, consistently with previous research [11,15,53] and similarly to findings in sheep meat [54,55]. Beyond its central role in protein synthesis, lysine is actively involved in vital physiological functions, including the generation of enzymes, hormones, and antibodies, supporting immune system efficiency, metabolic processes, and collagen formation essential for the integrity of skin, bones, and connective tissues [56].

Similarly, to sheep meat, among the non-essential amino acids, glutamine and aspartic acid are the most represented, aligning with data reported for Italian donkey and equine meat [11,12,14,53,55]. Additionally, a considerable amount of arginine is observed for two types of meat, which play important roles in vascular homeostasis, spermatogenesis, and fetal growth. Arginine is considered conditionally essential when endogenous synthesis is insufficient to meet metabolic needs, often occurring during children’s growth and in highly catabolic conditions [57,58].

Differences in amino acid composition have emerged, although this result did not affect the *EAA*/*AAT* percentages, which were comparable for both species, highlighting the high nutritional value of equine meat. Essential amino acids are fundamental in the diet, especially for certain population groups with specific needs, such as children, the elderly, and the sick.

### 3.4. Fatty Acid Composition of Raw Donkey and Horse Meat

The results obtained for the meat fatty acid profile of donkey and horse meat are presented in Table 4.

Horse meat exhibited a significantly higher total lipid content (*p* < 0.01), along with greater amounts of saturated fatty acids (SFAs, *p* < 0.01), particularly myristic, palmitic, and stearic acids (*p* < 0.001), compared to donkey meat. It also contained more monounsaturated fatty acids (MUFAs, *p* < 0.001) but had lower polyunsaturated fatty acids (PUFAs, *p* < 0.001). In terms of nutritional indices, horse meat had lower PUFA/SFA (*p* < 0.01) and n-6/n-3 ratios (*p* < 0.001), while showing higher values for the atherogenic index (*AI*, *p* < 0.05) and thrombogenic index (*TI*, *p* < 0.05) compared to donkey meat.

The distinct fatty acid profiles in equine muscles played a partial role in shaping these health-related lipid indices. Nutritionally, it is noteworthy that the n-6/n-3 ratio in horse meat is close to the World Health Organization’s (WHO) recommended limit of 4.0, beyond which there is an increased risk of atherosclerosis and cardiovascular issues [24]. Conversely, donkey meat had slightly higher n-6/n-3 values than the WHO’s recommended threshold [11,15], which may be due to the greater concentrations of linoleic acid (C18:2) and arachidonic acid (C20:4) found in donkey meat compared to horse meat.

The atherogenic and thrombogenic indices in both horse and donkey muscles were comparable to those reported in earlier studies [11,13,26,59,60,61]. This highlights the importance of not only considering PUFA/SFA and n-6/n-3 ratios but also recognizing the differing metabolic impacts of individual saturated and polyunsaturated fatty acids when assessing the nutritional value of meat [62]. Different fatty acids can exert varying influences on the development or prevention of atherosclerotic and thrombotic conditions [63].

In horse meat, saturated and monounsaturated fatty acids predominate, making up 32.44% and 39.58%, respectively, while donkey meat contains a higher proportion of PUFAs (31.51%). These findings are consistent with previous research [11,12,13,15]. However, the fatty acid profiles of horse and donkey meat can vary considerably, largely due to differences in farming practices and the age of the animals at slaughter [13,64]. The elevated PUFA content in donkey meat compared to horse meat may be attributed to the species’ leaner nature. In leaner animals, phospholipids, which are more unsaturated than triacylglycerols, contribute more significantly to the meat’s fatty acid profile, whereas triacylglycerol levels rise in animals with higher total lipid content [11,21,22,35].

In comparison, sheep meat has a higher saturated fatty acid (SFA) content, ranging between 51% and 55%, while the concentration of polyunsaturated fatty acids (PUFAs) does not exceed 11%. Additionally, the thrombogenic index (*TI*) and atherogenic index (*AI*) in sheep meat are both above 1, indicating a higher risk of blood clot formation and the development of atherosclerosis. The PUFA n-6-to-n-3 ratio (n-6/n-3) in sheep meat is also less favorable, not exceeding [46,65]. In contrast, horse and donkey meats are notable for their healthier fatty acid profile. They contain lower levels of SFAs, which are often linked to an increased risk of cardiovascular diseases when consumed in excess. Equid meat also has a higher concentration of PUFAs, which are beneficial for heart health due to their anti-inflammatory properties and their ability to lower cholesterol levels. The n-6/n-3 is more favorable in equid meat, supporting better cardiovascular protection and anti-inflammatory effects. Moreover, equid meat presents lower levels of TI and AI, making it a healthier option compared to sheep meat.

The quantitative and qualitative chemical composition and physicochemical proprieties of donkey kaddid and horse kaddid are presented in Table 5.

The donkey kaddid exhibits a higher protein content (17.45 g/100 g) than the horse kaddid (16.7 g/100 g), making it a richer source of protein. This indicates that donkey kaddid could be a more valuable option for those seeking higher protein intake. As for sheep kaddid meat, the protein content decreases significantly (*p* < 0.05) during the drying process [59,60,61,65]. This reduction can be attributed to the partial denaturation of proteins due to water loss and changes in chemical bonds within protein molecules during drying [65]. The amino acid profile for donkey and horse kaddid was comparable to results for raw meat. The drying process of meat significantly affects methionine composition due to the sensitivity of this amino acid to salt and high temperatures [59].

Relative to the mineral content in raw meat, both salting and drying processes result in a higher mineral content. This increase occurs because drying evaporates the water from the meat, thereby concentrating the minerals, such as salts and ash. The salting process also introduces more salt, raising the mineral content. After drying and salting, both types of meat display significant variations in all color parameters [66]. These alterations in color result from the drying process, which concentrates pigments and reduces moisture, and the salting process, which affects color through chemical interactions between salts and the meat’s intrinsic pigments. Consequently, there are marked differences in hue, intensity, and overall appearance of the meat [13,21,33]. This phenomenon is similarly observed in kaddid sheep meat [59,65].

**Figure 1:**
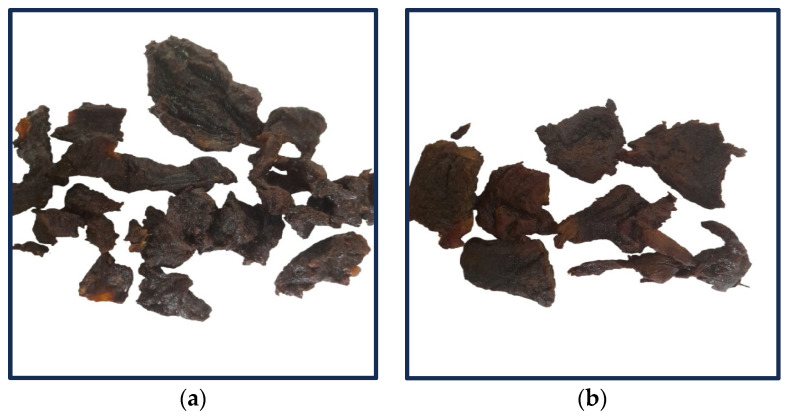
The external appearance of (**a**) donkey kaddid and (**b**) horse kaddid.

Horse kaddid (Figure 1b) shows a significantly higher fat content than donkey kaddid (Figure 1a) (*p* < 0.05). The increase in fat content post-drying can be explained by the infiltration of melted fat into the muscle tissue during the drying process and the alteration of the muscle structure due to the heat applied during drying, which facilitates the extraction of fat [67].

Horse kaddid has higher saturated fatty acids (SFAs) and monounsaturated fatty acids (MUFAs). Donkey kaddid is characterized by a higher proportion of polyunsaturated fatty acids (PUFAs). This distinction is crucial as PUFAs are often associated with various health benefits, including improved cardiovascular health. The PUFA/SFA ratio is also more favorable in donkey kaddid, suggesting a potentially healthier fatty acid composition. Data from the literature show that kaddid sheep meat has a higher saturated fatty acid (SFA) content, ranging from 44% to 51%, while the concentration of polyunsaturated fatty acids (PUFAs) does not exceed 9% [65]. In contrast, kaddid horse and donkey meats are notable for their healthier fatty acid profiles, offering better cardiovascular protection and anti-inflammatory effects. Similar observations were noted for both species. Compared to raw donkey and horse meat, the protein levels in kaddid donkey and horse meat were significantly higher, while the fat and ash content were significantly lower (*p* < 0.05). Regarding color parameters, kaddid donkey and horse meat exhibited significant variations for all measured parameters (*p* < 0.05), with kaddid meats being less luminous and tending toward black compared to raw donkey and horse meats. Donkey meat had lower levels of palmitic acid (C16:0) and stearic acid (C18:0) but higher levels of oleic acid (C18:1) and linoleic acid (C18:2) compared to kaddid donkey, with similar results observed for horse meat. Only the saturated fatty acid (SFA) content varied between the two types of meat. For the amino acid profile, the methionine content differed significantly between raw and kaddid donkey meat, and the total amino acid content was significantly higher in raw donkey and horse meats compared to their kaddid counterparts.

The drying process significantly affects the levels of saturated fatty acids such as palmitic acid (C16:0) and stearic acid (C18:0). This effect is due to the dehydration process, which increases the apparent concentration of certain components, including these saturated fatty acids. The reduction in overall mass due to water loss makes the remaining fat content more concentrated. Additionally, there is a significant decrease in the unsaturated fatty acids oleic acid (C18:1) and linoleic acid (C18:2), which leads to an increased PUFA/SFA ratio in the kaddid, attributed to lipid [59] oxidation [67].

## 4. Conclusions

The physico-chemical characterization of equine meat, encompassing both donkey and horse meat, is paramount for assessing its nutritional quality. The results of this study highlight significant differences in the physico-chemical properties, amino acid profiles, and fatty acid composition between North African donkey meat and Arab-Barb horse meat. Donkey meat, with its higher protein content and lower fat content, may represent a more advantageous source of essential proteins, particularly appealing to consumers concerned with fat intake. The amino acid analysis revealed that both types of meat provide all essential amino acids necessary for adequate nutrition, which is particularly beneficial for specific population groups such as children and the elderly. Additionally, donkey meat showed a higher percentage of polyunsaturated fatty acids (PUFAs), while horse meat contained higher levels of saturated and monounsaturated fatty acids (SFAs and MUFAs), affecting health-related lipid indices differently. These findings suggest that both donkey and horse meat offer distinct nutritional benefits, influenced by factors such as breed, feeding practices, and the conditions under which the animals are raised. In comparison with lamb meat, traditionally used in the production of kaddid, donkey and horsemeat provide notable health advantages. Lamb meat is characterized by its higher fat content, particularly its elevated levels of saturated fatty acids (SFAs) and relatively low proportion of PUFAs. This higher SFA content in lamb contributes to elevated atherogenic index (*AI*) and thrombogenic index (*TI*), indicating a higher potential risk for cardiovascular issues. Furthermore, the n-6/n-3 ratio in lamb is less favorable compared to equid meat. The leaner fat profile of donkey and horse meat, coupled with their higher PUFAs content and more favorable n-6/n-3, positions them as healthier alternatives to lamb in kaddid production. Consequently, the inclusion of donkey or horse meat in kaddid offers not only a healthier alternative to lamb but also a continuation of culinary tradition with enhanced nutritional benefits.

## Figures and Tables

**Table 1:** Carcass characteristics of donkey and horse breeds (means ± SEM).

Performance	North African Donkey Population	Horse Arab Barb
Live weight (kg)	205.1 ± 6.1 ^b^	298.6 ± 14.2 ^a^
Cold carcass weight (kg)	109.9 ± 3.6 ^b^	193.9 ± 6.4 ^a^
Cold dressing (%)	53.5 ± 2.3 ^b^	64.9 ± 4.2 ^a^

Different letters (a, b) indicate significant differences at *p* < 0.05.

**Table 2:** Chemical and physico-chemical parameters of *Longissimus thoracis* from and North African donkey and Arab-Barb horse (means ± SEM).

	Raw Donkey Meat	Raw Horse Meat
Protein (g/100 g)	22.1 ± 0.50 ^a^	20.84 ± 0.52 ^b^
Fat (g/100 g)	1.23 ± 0.24 ^b^	2.09 ± 0.82 ^a^
Ash (g/100 g)	1.10 ± 0.09	0.95 ± 0.12
pH_24h_	5.90 ± 0.10	6.10 ± 0.18
Moisture (g/100 g)	75.7 ± 0.53	76.12 ± 0.50
*CL* (%)	43.54 ± 0.93	42.20 ± 1.28
*WHC* (%)	85.32 ± 0.54	84.82 ± 0.63
Color Parameters		
L*	39.62 ± 0.54 ^a^	32.45 ± 0.92 ^b^
a*	15.32 ± 0.72 ^b^	16.82 ± 1.14 ^a^
b*	0.64 ± 0.25 ^b^	3.80 ± 0.84 ^a^
c*	17.24 ± 0.32	16.22 ± 1.10
h*	0.45 ± 0.02 ^b^	13.86 ± 0.73 ^a^

Different letters (a, b) indicate significant differences at *p* < 0.05.

**Table 3:** Amino acid composition (g/100 g) of North African donkey and Arab-Barb horse raw meat (means ± SEM).

	Raw Donkey Meat	Raw Horse Meat	
Aspartic acid	1.61 ± 0.55	1.70 ± 0.62	
Glutamine	3.24 ± 0.52 ^a^	2.89 ± 0.42 ^b^	
Serine	0.50 ± 0.20	0.48 ± 0.23	
Glycine	0.71 ± 0.26	0.68 ± 0.24	
Alanine	0.87 ± 0.19	0.75 ± 0.22	
Tyrosine	0.66 ± 0.12	0.50 ± 0.08	
Proline	0.54 ± 0.10 ^b^	0.88 ± 0.12 ^a^	
Histidine	0.56 ± 0.06 ^b^	0.84 ± 0.08 ^a^	
Threonine	1.06 ± 0.32	0.71 ± 0.25	
Arginine	1.34 ± 0.20	1.15 ± 0.18	
Valine	0.48 ± 0.14	0.57 ± 0.13	
Methionine	1.08 ± 0.09 ^a^	0.82 ± 0.07 ^b^	
Phenyalanine	0.60 ± 0.05	0.65 ± 0.07	
Isoleucine	0.92 ± 0.08 ^a^	0.78 ± 0.07 ^b^	
Leucine	1.53 ± 0.14 ^a^	1.25 ± 0.12 ^b^	
Lysine	2.27 ± 0.32 ^b^	3.10 ± 0.44 ^a^	
Total *AAT*	18.05 ± 0.55	17.82 ± 0.60	
*EAA*	8.50 ± 0.25	8.72 ± 0.32	
*EAA*/*AAT* (%)	47.05 ± 0.85	48.9 ± 0.95	

*AAT*—total amino acids; *EAA*—essential amino acids; different letters (a, b) indicate significant differences at *p* < 0.05.

**Table 4:** Fatty acid composition (% total fatty acids) of North African donkey and Arab-Barb horse raw meat (means ± SEM).

	Raw Donkey Meat	Raw Horse Meat
C12:0	0.25 ± 0.04 ^b^	0.49 ± 0.03 ^a^
C14:0	2.98 ± 0.26 ^b^	4.60 ± 0.28 ^a^
C14:1	0.32 ± 0.05	0.34 ± 0.04
C15:0	0.44 ± 0.09	0.39 ± 0.05
C15:1	1.40 ± 0.06	1.38 ± 0.06
C16:0	24.56 ± 0.52 ^b^	29.52 ± 0.84 ^a^
C16:1	4.12 ± 0.28 ^b^	9.21 ± 0.35 ^a^
C18:0	7.02 ± 0.32 ^a^	4.06 ± 0.45 ^b^
C20:1 n-6	0.35 ± 0.09	0.33 ± 0.07
C18:1 n-9	26.25 ± 0.59 ^b^	29.74 ± 0.78 ^a^
C18:2 n-6	22.4 ± 0.45 ^a^	14.89 ± 0.36 ^b^
C18:3 n-3	2.99 ± 0.22	3.14 ± 0.10
C20:2 n-6	0.06 ± 0.02	0.04 ± 0.02
C20:3 n-3	0.36 ± 0.03 ^a^	0.05 ± 0.01 ^b^
C20:4 n-6	5.21 ± 0.22 ^a^	2.54 ± 0.25 ^b^
C20:5 n-3	0.17 ± 0.02 ^a^	0.04 ± 0.01 ^b^
C22:2 n-6	0.15 ± 0.03	0.17 ± 0.05
C22:6 n-3	0.17 ± 0.02	0.18 ± 0.02
SFAs	36.05 ± 0.56 ^b^	39.37 ± 0.54 ^a^
MUFAs	32.44 ± 1.25 ^b^	39.58 ± 1.65 ^a^
PUFAs	31.51 ± 1.02 ^a^	21.05 ± 1.45 ^b^
PUFA/SFA	0.87 ± 0.07 ^a^	0.53 ± 0.02 ^b^
n-3	3.69 ± 0.20	3.41 ± 0.31
n-6	28.17 ± 1.10 ^a^	17.97 ± 1.36 ^b^
n-6/n-3	7.63 ± 0.89 ^a^	5.26 ± 0.74 ^b^
*AI*	0.49 ± 0.06 ^b^	0.63 ± 0.05 ^a^
*TI*	0.83 ± 0.05 ^b^	0.97 ± 0.02 ^a^

Different letters (a, b) indicate significant differences at *p* < 0.05.

**Table 5:** Quantitative and qualitative chemical composition and physicochemical properties of donkey and horse kaddid (means ± SEM).

	Donkey Kaddid	Horse Kaddid	Drying Process Effect
Protein (g/100 g)	17.45 ± 0.43 ^a^	16.7 ± 0.35 ^b^	**
Fat (g/100 g)	1.45 ± 0.14 ^b^	2.85 ± 0.11 ^b^	*
Ash (g/100 g)	1.64 ± 0.12	1.56 ± 0.15	*
pH	6.20 ± 0.20	6.30 ± 0.16	NS
**Color Parameters**			
L*	31.42 ± 0.65 ^a^	27.38 ± 0.56 ^b^	*
a*	11.24 ± 0.42 ^b^	13.52 ± 0.86 ^a^	*
b*	0.34 ± 0.12 ^b^	2.30± 0.65 ^a^	*
c*	11.47 ±0.33 ^b^	13.72 ± 0.72 ^a^	*
h*	1.72 ± 0.25 ^b^	9.65 ± 1.12 ^a^	*
**Fatty acids** (% total fatty acids)			
C12:0	0.22 ± 0.05 ^b^	0.48 ± 0.04 ^a^	NS
C14:0	2.92 ± 0.24 ^b^	4.65 ± 0.26 ^a^	NS
C14:1	0.32 ± 0.06	0.33 ± 0.05	NS
C15:0	0.47 ± 0.09	0.39 ± 0.05	NS
C15:1	1.39 ± 0.06	1.38 ± 0.06	NS
C16:0	26.80 ± 0.61 ^b^	30.44 ± 0.77 ^a^	**
C16:1	4.12 ± 0.28 ^b^	9.21 ± 0.35 ^a^	NS
C18:0	8.32 ± 0.33 ^a^	4.86 ± 0.39 ^b^	**
C20:1 n-6	0.35 ± 0.09	0.33 ± 0.07	NS
C18:1 n-9	24.2 ± 0.43 ^b^	27.98 ± 0.62 ^a^	**
C18:2 n-6	21.95 ± 0.56 ^a^	13.97 ± 0.88 ^b^	*
C18:3 n-3	2.86 ± 0.29	2.93 ± 0.21	NS
C20:2 n-6	0.06 ± 0.02	0.04 ± 0.02	NS
C20:3 n-3	0.32 ± 0.03 ^a^	0.07 ± 0.01 ^b^	NS
C20:4 n-6	5.18 ± 0.26 ^a^	2.52 ± 0.26 ^b^	NS
C20:5 n-3	0.18 ± 0.02 ^a^	0.06 ± 0.01 ^b^	NS
C22:2 n-6	0.16 ± 0.04	0.17 ± 0.05	NS
C22:6 n-3	0.18 ± 0.02	0.19 ± 0.02	NS
SFAs	38.73 ± 0.82 ^b^	40.82 ± 0.56 ^a^	*
MUFAs	30.38 ± 0.69 ^b^	39.23 ± 0.52 ^a^	NS
PUFAs	30.89 ± 0.49 ^a^	19.95 ± 0.38 ^b^	NS
PUFAs/SFAs	0.79 ± 0.15 ^a^	0.48 ± 0.12 ^b^	NS
**Amino acids (** **g/100 g)**			
Aspartic acid	1.63 ± 0.52	1.68 ± 0.56	NS
Glutamine	3.10 ± 0.50 ^a^	2.79 ± 0.42 ^b^	NS
Serine	0.51 ± 0.18	0.46 ± 0.23	NS
Glycine	0.71 ± 0.24	0.69 ± 0.26	NS
Alanine	0.83 ± 0.18	0.73 ± 0.24	NS
tyrosine	0.66 ± 0.10	0.52 ± 0.10	NS
Proline	0.55 ± 0.10 ^b^	0.84 ± 0.15 ^a^	NS
Histidine	0.46 ± 0.04 ^b^	0.61 ± 0.05 ^a^	NS
Threonine	1.05 ± 0.29	0.79 ± 0.26	NS
Arginine	1.14 ± 0.30	1.08 ± 0.22	NS
Valine	0.48 ± 0.12	0.55 ± 0.14	NS
Methionine	0.88 ± 0.07 ^a^	0.72 ± 0.04 ^b^	*
Phenyalanine	0.60 ± 0.05	0.62 ± 0.06	NS
Isoleucine	0.93 ± 0.08 ^a^	0.73 ± 0.09 ^b^	NS
Leucine	1.51 ± 0.16 ^a^	1.24 ± 0.13 ^b^	NS
Lysine	1.88 ± 0.26 ^b^	2.56 ± 0.31 ^a^	NS
Total *AAT*	16.96 ± 0.42	16.61 ± 0.60	**
*EAA*	7.79 ± 0.32	7.82 ± 0.39	*
*EAA/AAT* (%)	45.93 ± 0.52	47.08 ± 0.61	*

Different letters (a, b) indicate significant differences at *p* < 0.05. * (*p* < 0.05); ** (*p* < 0.01). NS: not significant.

## Data Availability

The original contributions presented in the study are included in the article, further inquiries can be directed to the corresponding author.
